# Successful Combination Therapy of Trazodone and Fluvoxamine for Pica in Alzheimer's Disease: A Case Report

**DOI:** 10.3389/fpsyt.2021.704847

**Published:** 2021-07-01

**Authors:** Tadashi Kanamori, Yoshiyuki Kaneko, Kouju Yamada, Masahiro Suzuki

**Affiliations:** ^1^Department of Psychiatry, Nihon University School of Medicine, Tokyo, Japan; ^2^Medical Department, Kunpukai Yamada Hospital, Tokyo, Japan

**Keywords:** Alzheimer's disease, case report, fluvoxamine, Klüver–Bucy syndrome, pica, trazodone

## Abstract

Pica in Alzheimer's disease (AD) makes it difficult for caregivers to provide care. However, few effective medications have been reported for pica in AD. We report a case of AD with pica that was successfully improved by trazodone and fluvoxamine. An 80-year-old woman with AD was admitted to our hospital due to aggravated pica, including eating weeds in the facility's garden and eating a dishwashing sponge. Her pica was accompanied by oral tendency, prosopagnosia, and placidity. She took rivastigmine and memantine, but these were ineffective for her pica. She was given olanzapine and perospirone, but both were discontinued due to over-sedation and severe extrapyramidal symptoms, respectively. We then administered trazodone and fluvoxamine, both of which have demonstrated effectiveness for pica in frontotemporal dementia (FTD). Her pica behaviors then disappeared without daytime sleepiness. In this case, pica with oral tendency, which was accompanied by prosopagnosia and placidity, may be interpreted as a partial symptom of Klüver–Bucy syndrome (KBS). KBS is often seen in FTD, but also occurs in late-stage AD. Our case together with previous reports showing that trazodone and fluvoxamine were effective for pica in FTD suggest that the same common drug therapy may be successful in pica with oral tendency, regardless of the subtype of dementia.

## Introduction

Pica is defined as the persistent eating of non-nutritive, non-food substances. It is well-known that iron deficiency anemia can cause pica, but pica is also found in psychiatric conditions such as dementia, intellectual disability, autism, and schizophrenia (1). Among degenerative dementias, frontotemporal dementia (FTD) is the most likely to have pica, but pica is also seen in 10% of patients with AD (2), making it difficult for caregivers to provide care. In FTD, pica can appear from the early stage, whereas in AD, it generally emerges in the late stage (3). Pica can sometimes cause life-threatening events, such as asphyxia and aspiration (4). Therefore, effective treatments for this condition in degenerative dementia are greatly needed.

Although the etiology of pica in degenerative dementia remains unclear, multiple factors are thought to be involved (5). It has been reported in the literature that pica in degenerative dementia appears in association with an oral tendency, a symptomatology of “putting the object into the mouth, biting gently, chewing, licking, touching with the lips” (6). The symptoms other than oral tendency that are involved in pica vary depending on the type of dementia. In FTD, binge eating and altered food preferences are also thought to be involved (7). In AD, it has been surmised that cognitive impairment, agnosia, and changes in taste and smell (8) are related to this condition.

Since the cause of pica in degenerative dementia has not been elucidated, there are only a few known effective drug therapies for this condition. In FTD, the effectiveness of some antidepressants such as trazodone (9) and fluvoxamine (10) has been demonstrated. In AD, antipsychotic drugs such as haloperidol were reported to be effective against pica (11). However, antipsychotics are associated with an increased risk of extrapyramidal symptoms and sedation. Since pica itself can be a risk factor for asphyxia and aspiration, effective treatments that do not cause sedation and extrapyramidal symptoms need to be explored for pica in patients with AD.

The oral tendency is assumed to be the common pathological feature of pica in FTD and AD. Therefore, in AD patients having an oral tendency, effective treatments reported for pica in FTD, such as antidepressants (9, 10), could also be effective for their pica. A case of AD with pica that was successfully improved by trazodone in combination with fluvoxamine is reported.

## Case Description

**Figure 1:**
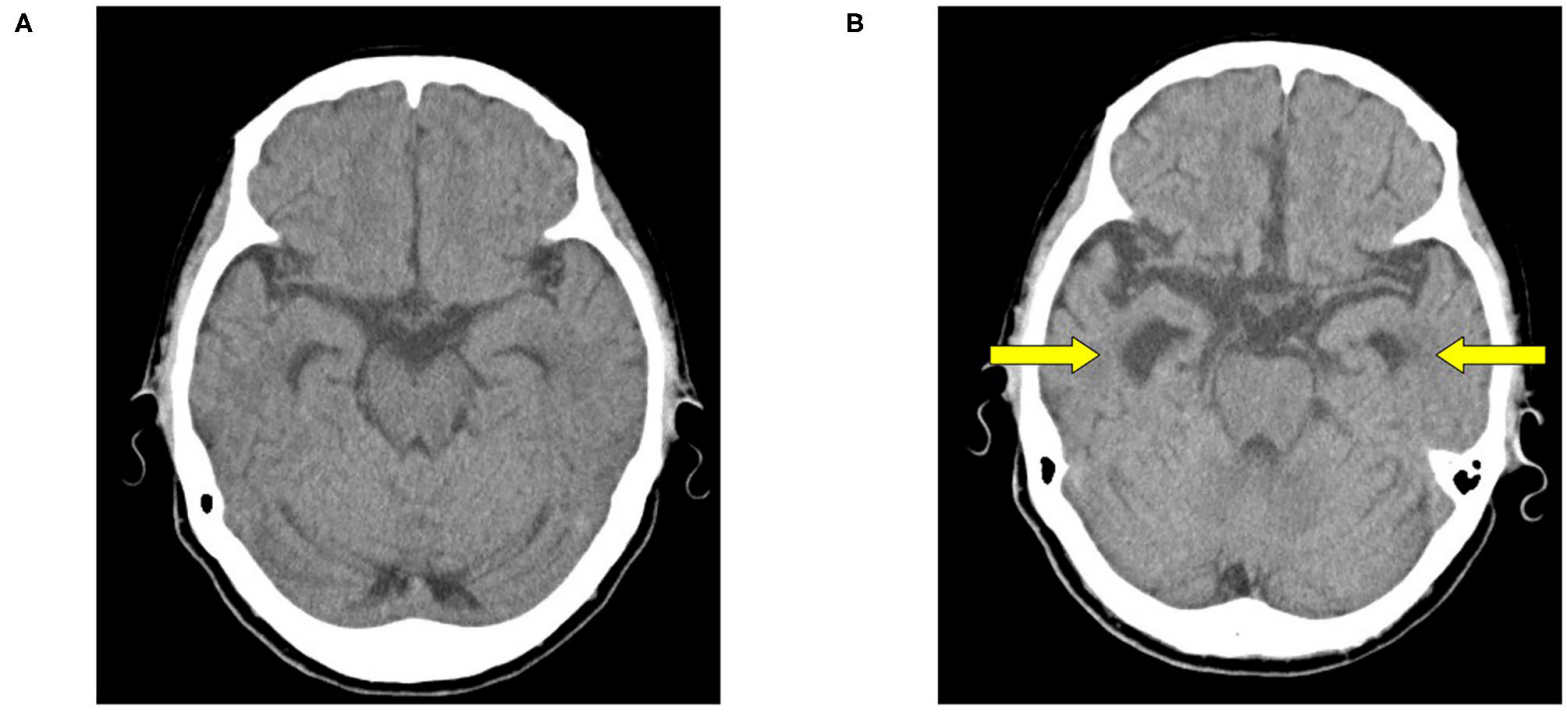
Head computed tomography (CT). **(A)** Age 77 years. **(B)** Age 80 years. At 3-year follow-up **(B)** head CT shows selective hippocampal atrophy, but no frontal or temporal lobe lateral atrophy.

An 80-year-old woman with AD was admitted to our hospital due to aggravated pica. The patient had no remarkable medical history. She had developed cognitive impairment at the age of 76 years, and visited our hospital the next year. Psychological testing with the revised Hasegawa's Dementia Scale (HDS-R) showed that her score was 20 of a total of 30 (the cut-off point is 20) (12), and she was unable to replicate the cube in the cube-copying test. Head computed tomography (CT) showed selective atrophy of the hippocampus (Figure 1A). We diagnosed her with AD and began rivastigmine. One year after the first visit to our hospital, she developed prosopagnosia and wandering, and was admitted to a nursing home. Seven months after entering the facility, she started to show abnormal behaviors of putting toilet paper into her mouth and eating pencil shavings. Furthermore, she exhibited an oral tendency of repeatedly licking metal rods. At the age of 80 years, rivastigmine was stopped, and memantine therapy was begun. However, her pica worsened, including eating weeds in the facility's garden and eating a dishwashing sponge. She was then admitted to our hospital.

Her psychiatric symptoms were mainly amnesia with no depression or compulsive behavior. She had no typical symptoms of FTD, such as behavioral disinhibition or stereotyped behavior. She was considered to be placid because she lacked emotional reactions such as anger and fear and was obedient to the instructions of facility staff. She was unable to cooperate with psychological tests, including the HDS-R. Blood tests showed a serum iron level of 89 μg/dL (normal range 40–180 μg/dL), unsaturated iron binding capacity of 190 μg/dL (normal range 150–385 μg/dL), and ferritin of 43.2 ng/mL (normal range 4.0–87.0 ng/dL). These results did not suggest iron deficiency, a major cause of pica. Head CT showed selective hippocampal atrophy, which had progressed compared with 3 years earlier (Figure 1B). Her medial temporal atrophy seemed asymmetrical (R>L), which raised the possibility of right temporal variant FTD. In right temporal variant FTD, prosopagnosia, abnormal behavior, and loss of empathy are seen. However, in this case, the initial symptoms were memory impairment and execution impairment, and abnormal behaviors, such as egocentrism and disinhibition, did not appear even in the later stages of the course. These suggest AD more than right temporal variant FTD (13).

We concluded that her pica was aggravated by the progression of Alzheimer's disease. After admission, she ate her diaper and drank water that others had spit out after brushing their teeth. The frequency of her pica was three times a week. In addition to 20 mg memantine, she was given 1.25 mg olanzapine and 4 mg perospirone, but both were discontinued due to over-sedation and severe extrapyramidal symptoms, respectively.

After receiving approval from the patient and her family for off-label use, trazodone, which is effective against pica in the management of FTD, was given (9). The frequency of pica decreased to once a week with a regimen of 50 mg trazodone once a day; however, biting a toothpaste tube was still observed. Increasing the amount of trazodone was expected to cause sleepiness. Therefore, fluvoxamine 75 mg, which is also effective for pica in FTD, was added (10). Her pica behaviors were then no longer observed. She was able to participate in occupational therapy without daytime sleepiness.

## Discussion

In this case, the combination of trazodone and fluvoxamine stopped pica behaviors in late-stage AD without the side effects of sedation and extrapyramidal symptoms. Few medication therapies are effective for treating pica in AD, which often appears in the late stage. In clinical practice, antipsychotics are often used to treat pica in AD (11), but these can cause sedation or aspiration. The dose of trazodone that has been reported to be effective against pica in FTD is 150–300 mg once a day (9). However, this dose of trazodone increases the risk of sedation. Therefore, in this case, the aim was to minimize the risk of sedation by the combination therapy of low-dose trazodone and fluvoxamine. This is the first case in which medication therapy was demonstrated to improve pica in AD without causing sedation and extrapyramidal symptoms.

Pica in dementia is broadly divided into pica with oral tendency and pica without oral tendency, the latter of which is described as changes in eating, such as appetite and food preference (14). In this case, pica with oral tendency, which was accompanied by prosopagnosia and placidity, may be interpreted as a partial symptom of Klüver–Bucy syndrome (KBS). KBS is a pathological condition characterized by oral tendency, visual agnosia including prosopagnosia, placidity, altered dietary habits, hypermetamorphosis, and hypersexuality (6). KBS is associated with pica as a result of changes in oral tendency and altered dietary habits (15). KBS is often seen in FTD (16), but also has been reported in late-stage AD (2). Our case together with previous reports showing that trazodone and fluvoxamine were effective for pica in FTD (9, 10) suggests that the same common drug therapy may be successful in pica with oral tendency, regardless of the subtype of dementia. Trazodone and fluvoxamine may improve pica in FTD by affecting the serotonergic nervous system (9, 10). Trazodone, which has a serotonin 2A antagonistic effect, and fluvoxamine, which has a serotonin reuptake effect, are expected to have a synergistic effect. However, the detailed mechanism is unknown. Our case suggests that pica in AD could be improved with fluvoxamine and/or trazodone, especially when the pica shows oral tendency.

Since this is a report of a single case, the difference between the effects of trazodone monotherapy, fluvoxamine monotherapy, and combination therapy for pica in AD cannot be determined. In addition, the optimal dose of trazodone and fluvoxamine for combination therapy for pica in AD remains unclear. Future studies are needed to clarify whether combination therapies are superior to monotherapy.

We reported the first case of successful combination therapy with trazodone and fluvoxamine for pica in AD without the side effects of sedation and extrapyramidal symptoms. Our case suggests that trazodone and fluvoxamine may be effective for treatment of pica with oral tendency, regardless of the subtype of dementia. The relationship between oral tendency and effectiveness of antidepressants on pica in AD should be elucidated in future research.

## Data Availability Statement

The original contributions presented in the study are included in the article/Supplementary Material, further inquiries can be directed to the corresponding author/s.

## Ethics Statement

Ethical review and approval was not required for the study on human participants in accordance with the local legislation and institutional requirements. The patients/participants provided their written informed consent to participate in this study. Written informed consent was obtained from the individual(s) for the publication of any potentially identifiable images or data included in this article.

## Author Contributions

TK and KY were involved in patient care and treatment. TK and MS mainly wrote the manuscript. KY and YK interpreted the patient data and were involved in revisions to the manuscript. All authors have read and approved the final version of the manuscript.

## Conflict of Interest

The authors declare that the research was conducted in the absence of any commercial or financial relationships that could be construed as a potential conflict of interest.
