# Supplementary material for: Successful Combination Therapy of Trazodone and Fluvoxamine for Pica in Alzheimer's Disease: A Case Report
Source: Front Psychiatry. 2021 Jul 1;12:704847. doi: 10.3389/fpsyt.2021.704847 (PMC8281115; doi:10.3389/fpsyt.2021.704847)
Supplement: Supplementary file 1 [file Table_1.DOCX]

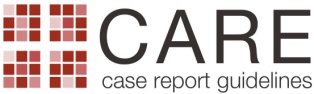
CARE Checklist of information to include when writing a case report
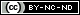


**Topic Item Checklist item description Reported on Line**

**Title 1** The diagnosis or intervention of primary focus followed by the words “case report” title

**Key Words 2** 2 to 5 key words that identify diagnoses or interventions in this case report, including "case report" 　11-12

Abstract

**(no references)**

**3a** Introduction: What is unique about this case and what does it add to the scientific literature? 14-15

**3b** Main symptoms and/or important clinical findings 15-16

**3c** The main diagnoses, therapeutic interventions, and outcomes 18-23

**3d** Conclusion—What is the main “take-away” lesson(s) from this case? 25-28

**Introduction 4** One or two paragraphs summarizing why this case is unique (**may include references**) 31-35

**Patient Information 5a** De-identified patient specific information 39-40

**5b** Primary concerns and symptoms of the patient 46-47, 49-50

**5c** Medical, family, and psycho-social history including relevant genetic information 39-40

**5d** Relevant past interventions with outcomes 44-50

Clinical Findings

**Timeline**

**Diagnostic Assessment**

**Therapeutic Intervention**

**Follow-up and Outcomes**

1. Describe significant physical examination (PE) and important clinical findings 41-44, 51-54
2. Historical and current information from this episode of care organized as a timeline 40-50

**8a** Diagnostic testing (such as PE, laboratory testing, imaging, surveys). 41-44, 54-57

**8b** Diagnostic challenges (such as access to testing, financial, or cultural) 51-57

**8c** Diagnosis (including other diagnoses considered) 57-58

**8d** Prognosis (such as staging in oncology) where applicable N/A

**9a** Types of therapeutic intervention (such as pharmacologic, surgical, preventive, self-care) 58-60, 62-66

**9b** Administration of therapeutic intervention (such as dosage, strength, duration) 58-62

**9c** Changes in therapeutic intervention (with rationale) 61-62

**10a** Clinician and patient-assessed outcomes (if available) 62-66

**10b** Important follow-up diagnostic and other test results N/A

**10c** Intervention adherence and tolerability (How was this assessed?) 66

**10d** Adverse and unanticipated events 58-60

**Discussion 11a** A scientific discussion of the strengths AND limitations associated with this case report 70-74

**11b** Discussion of the relevant medical literature **with references** 75-87

**11c** The scientific rationale for any conclusions (including assessment of possible causes) 86-87

**11d** The primary “take-away” lessons of this case report (without references) in a one paragraph conclusion 88-92

**Patient Perspective 12** The patient should share their perspective in one to two paragraphs on the treatment(s) they received N/A

**Informed Consent 13** Did the patient give informed consent? Please provide if requested . . . . . . . . . . . . . . . . . . . . . . . . . . . . . . . . . . . . . . **Yes ✓ No**
